# Gender health care inequalities in health crisis: when uncertainty can lead to inequality

**DOI:** 10.1186/s13690-024-01276-7

**Published:** 2024-04-02

**Authors:** Isabel Aguilar-Palacio, Blanca Obón-Azuara, Sara Castel-Feced, Sara Malo, Julia Teresa, María José Rabanaque

**Affiliations:** 1 Department of Preventive Medicine and Public Health, University of Zaragoza, Zaragoza, Spain; 2 Grupo de Investigación en Servicios Sanitarios de Aragón (GRISSA), Fundación Instituto de Investigación Sanitaria de Aragón (IIS Aragón), Zaragoza, Spain; 3 Research Network On Chronicity, Primary Care and Health Promotion (RICAPPS), Carlos III Health Institute (ISCIII), Madrid, Spain; 4 Servicio de Medicina Intensiva. Hospital Clínico Universitario Lozano Blesa, Zaragoza, Spain; 5 Servicio Aragonés de Salud (SALUD), Zaragoza, Spain

**Keywords:** Gender inequalities, Health crisis, COVID-19, Health care use

## Abstract

**Background:**

In health crisis, inequalities in access to and use of health care services become more evident. The objective of this study is to analyse the existence and evolution of gender inequalities in access to and use of healthcare services in the context of the COVID-19 health crisis.

**Methods:**

Retrospective cohort study using data from all individuals with a confirmed COVID-19 infection from March 2020 to March 2022 in Aragón (Spain) (390,099 cases). Health care access and use was analysed by gender for the different pandemic waves. Univariate and multivariate analyses were conducted to evaluate the effect of sex in health care. Blinder-Oaxaca decomposition methods were performed to explain gender gaps observed.

**Results:**

The health care received throughout the COVID-19 pandemic differed between men and women. Women were admitted to hospital and intensive care units less frequently than men and their stays were shorter. Differences observed between men and women narrowed throughout the pandemic, but persisted even after adjusting for age, socioeconomic status, morbidity burden or the patient's place of residence. Differences in sociodemographic characteristics and morbidity burden could explain partially the gender inequalities found, mainly in the later phases of the pandemic, but not in the earlier waves.

**Conclusions:**

There were gender inequalities in access to and use of health services during the COVID-19 pandemic. Inequalities were greater in the first waves of the pandemic, but did not disappear. Analysis of health crises must take into account an intersectional gender perspective to ensure equitable health care.

**Supplementary Information:**

The online version contains supplementary material available at 10.1186/s13690-024-01276-7.

**Table:** 

Text box 1. Contributions to the literature
• Despite the importance of the gender approach to health crisis management, which involves considering gender inequalities in policy analysis, planning, design and implementation, it is not sufficiently applied
• We observed gender inequalities in the access to and use of health services during the COVID-19 pandemic. These inequalities were greater in the first waves of the pandemic, but did not disappear
• Preparing for future pandemics and health crises by focusing on care for the most vulnerable groups is necessary to avoid exacerbating existing inequalities

## Background

During health crisis there is a complex relationship between competing pathologies and the social and structural conditions in which social health inequalities are propagated and reinforced [1, 2]. In relation to gender, it is also during crises that large differences in mortality and vulnerability to disease become apparent [3]. But these inequalities are not limited to the risk of contracting the disease, but also to inequalities in access to and use of health care services [4].

The state of alarm generated during the COVID-19 pandemic, as has been described in other pandemics such as Zika or Ebola [5–7], had a differential effect on women. The likelihood of contracting pathologies increased, since women provided most of the informal care within families. As a result, they also experienced a higher frequency of work limitations and reduced income. All these situations mean a greater burden of illness for women for gender reasons, as they are exposed to a greater situation of vulnerability, which entails a differential vulnerability between men and women. This also limits their degree of freedom in decision-making and conditions the practices related to the pandemic that women and men have in their living environment, including their relationship with health care [8].

Although this is a well-known fact, some authors point out that during crisis health data are often incomplete and information is not always disaggregated by sex [9]. This makes it difficult to establish a relationship between sex and susceptibility to the disease, as well as in the health care received. Also, the impact on gender is not sufficiently taken into account, nor are health policy efforts and measures adopted from a public health perspective [10]. In this sense, the United Nations published a report [11] highlighting the importance and necessity of a gender approach in crisis management, which implies considering gender inequalities in the analysis, planning, design and implementation of policies, along with actions to address the different situations and needs that may arise.

To analyse the extent to which health emergencies affect women and men unequally is critical to develop effective and equitable policies and interventions in future crises. In this context, the aim of our study is to analyse the existence and evolution of gender inequalities in access to and use of healthcare services in the context of a health crisis, using the COVID-19 pandemic as a case study.

## Methodology

### Design, information sources and study population

We conducted a retrospective cohort study using data from the Aragón-COVID19 cohort. This is a health data collection of all individuals undergoing COVID-19 testing in the Spanish region of Aragón since March 2020. Aragón is an Autonomous Community located in the northeast of Spain. It is the fourth Spanish Community by extension but occupies the 11th place of 17 in terms of population. It has a population of 1.3 million inhabitants and half of the population live in the city of Zaragoza. Spain has a public health system which covers practically the entire population.

People included in the Aragón-COVID19 cohort were tested by the public health system either when they had symptoms compatible with COVID-19 or when they had close contact with a confirmed subject. All COVID-19 cases were confirmed using polymerase chain reaction (PCR) or COVID antigen testing. The Aragón-COVID19 cohort includes information gathered from administrative health data sources as well as electronic health records of the Aragón Healthcare Service.

All individuals in the cohort included in this study were those tested from 9 March 2020, the first epidemiological week with COVID-19 cases reported in Aragón, to 31 March 2022, which corresponded to the seventh wave in Aragón.

### Variables of the study

Of all the individuals in the cohort we analyzed their sociodemographic and clinical information, and their use of healthcare services related with the COVID-19 episode.

The main variable analysed was sex, obtained from the Users Database of Aragon Healthcare Service. Regarding sociodemographic characteristics, we also considered the age (under 15, 15–44, 45–64, 65–79 and 80 years or older), socioeconomic level and information about the place of residence. Socioeconomic level was calculated on the basis of pharmacy copayment levels and social security benefits received. From the combination of these two variables, 7 mutually exclusive categories were obtained: employed individuals earning less than €18,000 per year, employed individuals earning €18,000 per year or more, individuals receiving the unemployment allowance, individuals with a contributory pension of less than €18,000 per year and receiving free medicines, individuals with a contributory pension of €18,000 per year or more, individuals affiliated to the mutual insurance system for civil servants and other situations not previously considered. We considered also some variables related with the place of residence of the patient. Residing in a long-term care (LTC) facility was considered. We described deprivation index of the Basic Healthcare Area of residence categorized into four quartiles, from least (Q1) to most (Q4) deprived [12]. Finally, we considered if patient resided in a rural or urban area, according to the Aragon Government [13].

The clinical information included was obtained from the morbidity adjusted groups (GMA) [14]. This source of information considers all medical diagnoses available in primary healthcare and hospital discharge records (Minimum Basic Data Set of Hospital Discharges). We considered GMA information from January 2020 in order to know the health status prior to the COVID-19 diagnosis of the cohort individuals. The three variables analyzed from GMA were morbidity burden (obtained from the aggregation of the patient’s different diagnoses), the presence of chronic morbidities and the presence of respiratory illnesses.

We used different indicators to evaluate healthcare delivery. Firstly, we considered the time from the beginning of COVID-19 symptoms to diagnosis, with a range from -7 to 15 days, in those patients with symptoms. We also calculated the time from the COVID-19 diagnosis to hospital admission (ranging from -15 to 30 days). We analyzed hospital admission (yes/no) and intensive care units (ICU) admission (yes/no) in this hospitalization. As we do not have the diagnosis related to hospitalization, we considered a hospitalization to be related to COVID-19 when it happened between -15 and 30 days of COVID-19 diagnosis. We also calculated the length of hospital stay and the length of ICU stay.

### Analyses

All the analyses were stratified by pandemic waves in three groups: wave 1 (from March to 20 June 2020), waves 2 and 3 (from 21 June to 27 December 2020) and waves 4 to 7 (from 28 December 2020 to March 2022). We separated them according to the knowledge of COVID-19 management in each stage, as well as with the epidemiological situation. So, wave 1 is analysed separately due to the high level of uncertainty with diagnosis and clinical management. In waves 2 and 3 the management of disease was clearer, but there was no access to vaccines. Finally, in waves 4 to 7 there was already a COVID-19 vaccine available and the management of disease had improved.

First, we described sociodemographic and clinical characteristics of patients included in the study. We performed descriptive analyses of each health care indicator stratified by sex and by each of the stages of the COVID-19 pandemic, in order to identify differences by gender. To analyse the effect of sex on health care utilization, we performed univariate and multivariate analyses separately by each COVID-19 pandemic stage. To evaluate the association between sex and time from symptoms to confirmation and time from diagnostic to confirmation, linear regression analyses were performed. We conducted logistic regression models to study the influence of sex in hospital and ICU admissions. For the length of hospital stay and length of ICU stay, Poisson regression models were performed. All the analyses were adjusted by age, the presence of comorbidities, socioeconomic level and residence in a long-term care facility, in order to control for these variables.

Sex differences in health care attention were decomposed using Blinder-Oaxaca decomposition method. This method divides the mean outcome observed differences between men and women into two components: the explained component, that captures the differences in the outcomes explained by the independent variables, and the unexplained component, that also captures all potential effects of unobserved variables. [Oaxaca RL: Male–female wage differentials in urban labor markets. Int Econ Rev. 1973, 14 (3): 693–709. 10.2307/2525981.]. A twofold decomposition applying Oaxaca R library and reference regression coefficients were calculated from a pooled regression model [https://cran.r-project.org/web/packages/oaxaca/vignettes/oaxaca.pdf].

Analyses were conducted using R 4.1.3 (2022–03-10). All data were pseudonymized and the research protocol was approved by The Clinical Research Ethics Committee of Aragón (CEICA) (PI20/184).

## Results

From March 2020 to March 2022 390,099 confirmed positive cases of COVID-19 were diagnosed in Aragón. 52.3% of the cases were diagnosed in women.

The most frequent group of age was those from 16 to 44 years old. In the older age group (≥ 80 years), women accounted for a higher percentage. By socioeconomic level, the highest percentage in men was in those employed with salaries higher than 18,000€ per year (34.4%). In women, it belonged to those women employed earning less than 18,000€ per year (38.5%). According to the place of residence, more women lived in LTC facilities and a urban predominance was observed. Related to health status, women had a higher morbidity burden than men (*p* < 0.001). There were differences by sexes for all the diseases studied, with the only exception of chronic kidney disease. Men had a higher prevalence than women of diabetes, ischemic heart disease, stroke, hypertension, chronic obstructive pulmonary disease (COPD), cirrhosis and HIV/AIDS. On the contrary, women showed a higher prevalence of heart failure, depression and dementia (Table 1).

**Table 1:** Sociodemographic and clinical characteristics of COVID-19 confirmed positive cases by sex (march 2020-march 2022) in Aragón, Spain

	**Men (*N* = 186,455)**	**Women (*N* = 203,644)**	***p***
Age (years)^a^			< 0.001
< = 15	34,028 (18.2%)	32,573 (16.0%)	
16–44	74,331 (39.9%)	82,414 (40.5%)	
45–64	52,002 (27.9%)	55,854 (27.4%)	
65–79	17,022 (9.13%)	17,801 (8.74%)	
≥ 80	9072 (4.87%)	15,002 (7.37%)	
Socioeconomic level^a^			< 0.001
Employed < 18,000€ per year	62,249 (33.4%)	78,324 (38.5%)	
Employed ≥ 18,000€ per year	64,099 (34.4%)	49,319 (24.2%)	
Unemployed	5458 (2.93%)	8521 (4.18%)	
Pensioner < 18,000€ per year	23,736 (12.7%)	34,979 (17.2%)	
Pensioner ≥ 18,000€ per year	12,597 (6.76%)	9507 (4.67%)	
Mutualist	8319 (4.46%)	8200 (4.03%)	
Other	9997 (5.36%)	14,794 (7.26%)	
Residing in a long-term care facility^a^	4237 (2.27%)	7707 (3.78%)	< 0.001
Deprivation quartile^a^			0.031
1 (least deprivation)	52,969 (28.8%)	58,772 (29.1%)	
2	44,674 (24.3%)	49,222 (24.4%)	
3	37,291 (20.3%)	40,264 (20.0%)	
4 (highest deprivation)	48,779 (26.6%)	53,469 (26.5%)	
Zone of residence^a^			< 0.001
Rural	52,403 (28.5%)	54,131 (26.8%)	
Urban	131,310 (71.5%)	147,596 (73.2%)	
Morbidity burden^b^	3.36 (3.97)	4.01 (4.19)	< 0.001
Morbidity^a^			
Diabetes Mellitus	11,465 (6.49%)	9421 (4.83%)	< 0.001
Heart failure	1891 (1.07%)	2553 (1.31%)	< 0.001
Ischemic heart disease	4905 (2.78%)	2362 (1.21%)	< 0.001
Stroke	2415 (1.37%)	2312 (1.19%)	< 0.001
Hypertension	27,430 (15.5%)	28,907 (14.8%)	< 0.001
COPD	4557 (2.58%)	2565 (1.31%)	< 0.001
Chronic kidney disease	6212 (3.51%)	6980 (3.58%)	0.299
Cirrhosis	2754 (1.56%)	1985 (1.02%)	< 0.001
HIV/AIDS	429 (0.24%)	252 (0.13%)	< 0.001
Depression	8233 (4.66%)	21,944 (11.2%)	< 0.001
Dementia	1725 (0.98%)	4246 (2.18%)	< 0.001

*N* number, *p* statistical significance. ^a^Number (percentage). ^b^Mean (Standard Deviation). *COPD* chronic obstructive pulmonary disease

**Fig. 1:**
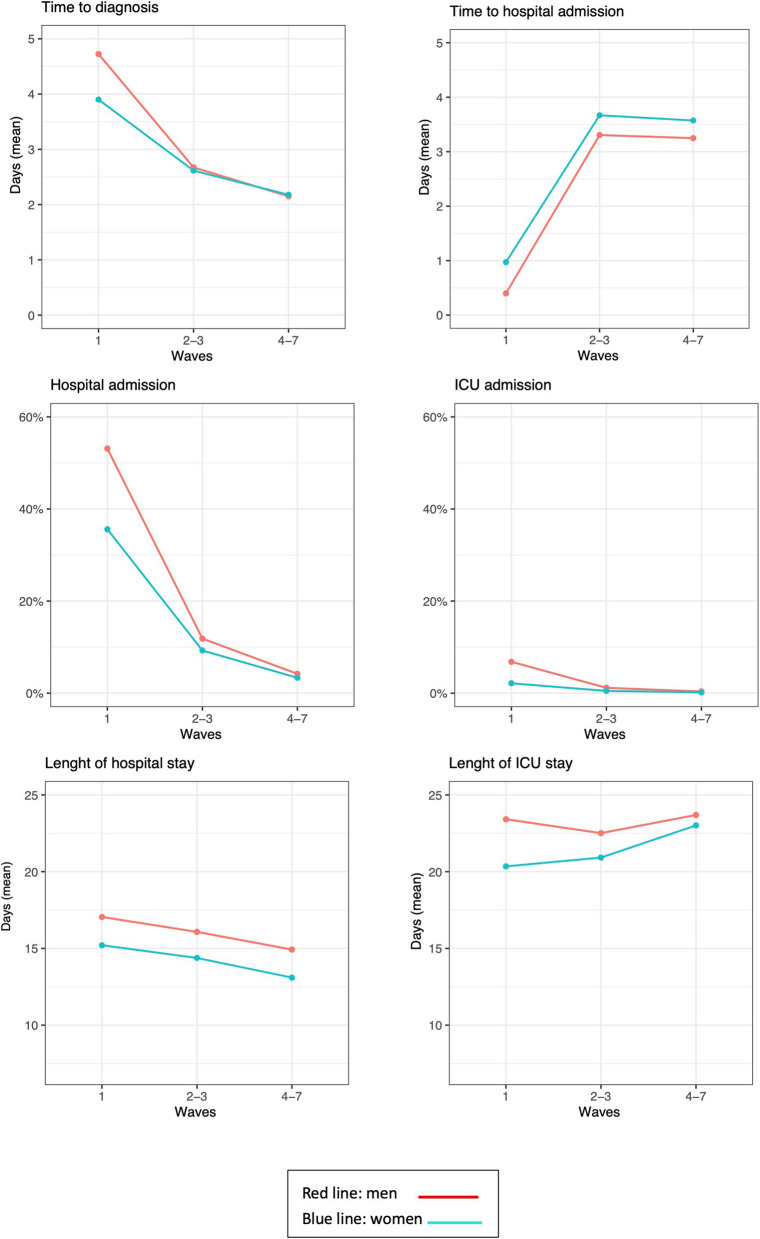
Evolution of health care indicators by sex and wave during the COVID-19 pandemic (march 2020-march 2022) in Aragón, Spain

We analysed the evolution across the COVID-19 pandemic of different health care indicators by sex. Time from symptoms to diagnosis was higher in men than in women during the first wave (mean: 4.7 days vs. 3.9; *p* < 0.001), but differences disappeared in following waves. On the contrary, time from diagnosis to hospital admission was lower in men than in women, and these differences remained for all the period (*p* = 0.001). The frequency of hospital admission and ICU admission was higher in men than in women especially in the first wave (53.1% vs 35.6% for hospital admission and 6.8% vs. 2.2% for ICU admission). These differences decreased in the following waves but persisted for all the pandemic (*p* < 0.001). Finally, length of hospital and ICU stay was higher in men than in women, and these differences persisted for the whole period analysed (Fig. 1).

We conducted univariate and multivariate models in order to know the effect of sex in the different health care indicators evaluated, adjusting by age, morbidity burden, socioeconomic level and residence in a long-term care facility (Table 2).

**Table 2:** Risk of women in relation to men for health care delivery by pandemic wave during the COVID-19 pandemic (march 2020-march 2022) in Aragón, Spain. Univariate and multivariate models

	**WAVE 1**				**WAVE 2–3**				**WAVE 4–7**			
	**Univariate model**		**Multivariate model**		**Univariate model**		**Multivariate model**		**Univariate model**		**Multivariate model**	
	**OR**	**95%CI**	**OR**	**95%CI**	**OR**	**95%CI**	**OR**	**95%CI**	**OR**	**95%CI**	**OR**	**95%CI**
Time to diagnosis (days)	-0.82	-1.09- -0.56	-0.63	-0.89 – -0.37	-0.06	-0.12–0.000	-0.05	-0.11–0.01	0.03	0.00–0.05	0.01	-0.01–0.04
Time to hospital admission (days)	0.57	0.28–0.87	0.53	0.23–0.84	0.36	0.13–0.60	0.55	0.30–0.79	0.32	0.13–0.52	0.52	0.31–0.72
Hospital admission	0.49	0.44–0.55	0.44	0.38–0.50	0.76	0.72–0.80	0.57	0.53–0.60	0.79	0.76–0.82	0.62	0.60–0.65
Lenght of hospital stay (days)	0.89	0.87–0.91	0.86	0.84–0.88	0.89	0.88–0.90	0.88	0.87–0.89	0.88	0.87–0.89	0.86	0.86–0.87
ICU admission	0.3	0.22–0.40	0.37	0.26–0.51	0.42	0.35–0.51	0.42	0.34–0.50	0.47	0.40–0.54	0.41	0.35–0.48
Lenght of ICU stay (days)	0.87	0.81–0.93	0.82	0.76–0.88	0.93	0.89–0.97	0.95	0.91–0.99	0.97	0.94–1.00	0.93	0.90–0.96

The time from symptom onset to diagnosis was shorter in women than in men in waves 1 and 2–3. Specifically, COVID-19 diagnosis in women showed a reduction of 0.82 days compared to men in wave 1. These differences decreased throughout the pandemic, showing no statistically significant differences in waves 2 to 7 (OR: 0.01, 95% CI: -0.01–0.04 in later waves). In contrast, the time from diagnosis to hospital admission was longer in women than in men. Thus, in women, the greatest differences were shown in the first wave, when they took 0.57 days longer to be admitted to hospital than men (95% CI: 0.28 to 0.87 days). These differences remained practically unchanged during the whole period analysed (OR: 0.52; 95% CI: 0.31 to 0.72 in the last waves).

The risk of hospital and ICU admission was lower in women than in men and remained statistically significant for the whole period, with higher differences in the risk of ICU admission (OR: 0.41; 95%CI: 0.35–0.48 in the last waves). Regarding the length of stay, it was lower in women than in men. In the case of length of hospital stay differences remained stable for all the period analysed (OR: 0.86; 95%CI 0.86–0.87 in the last waves). On the contrary, differences between men and women regarding length of ICU stay decreased across the pandemic, but remained statistically significant (OR: 0.82 in the first wave vs. 0.93 in the last waves).

Time to diagnosis and time to hospital admission: linear regression model; Hospital admission and ICU admission: logistic regression model; Length of hospital stay (days) and length ICU stay (days): Poisson model; Analyses adjusted by age, morbidity burden, socioeconomic level, area of residence (urban or rural) and residence in a long-term care facility.

In order to explain the gender gaps observed, Oaxaca decomposition analyses were conducted for each wave (Table 3). Variables included in the analyses were age, morbidity burden, socioeconomic level, area of residence (urban or rural) and residence in a LTC facility. Explained fraction of the gender gap increased across the pandemic for all the health care indicators analysed, with the only exception of length of hospital stay and length of ICU stay. The health care indicator with the highest explained estimate was time to diagnosis at waves 4–7 (52.8%) followed by hospital admission (31.11%).

**Table 3:** Decomposition of gender inequality in health care delivery by pandemic waves during the COVID-19 pandemic (march 2020-march 2022) in Aragón, Spain. Oaxaca decomposition analyses

	**Wave 1**		**Waves 2–3**		**Waves 4–7**	
	**Absolute**	**Relative (%)**	**Absolute**	**Relative (%)**	**Absolute**	**Relative (%)**
**Time to diagnosis**						
Explained	0.006	0.739	0.008	16.390	-0.015	52.789
Unexplained	0.794	99.261	0.043	83.610	-0.0137	47.211
**Time to hospital admission**						
Explained	0.004	0.699	0.135	21.072	0.145	24.266
Unexplained	-0.573	99.301	-0.507	78.928	-0.451	75.734
**Hospital admission**						
Explained	0.093	8.950	-0.174	25.955	-0.159	31.114
Unexplained	0.942	91.050	0.495	74.045	0.351	68.886
**Lenght of hospital stay**						
Explained	-0.938	23.840	-1.135	29.770	-0.507	17.756
Unexplained	2997	76.160	2678	70.230	2347	82.244
**ICU admission**						
Explained	0.087	5.879	-0.070	7.108	-0.141	14.318
Unexplained	1.393	94.121	0.916	92.892	0.846	85.682
**Lenght of ICU stay**						
Explained	-0.636	12.582	0.480	28.910	-0.253	17.523
Unexplained	4.418	87.418	1.180	71.090	1.190	82.477

Variables included in the Oaxaca decomposition analyses: age, morbidity burden, socioeconomic level, area of residence (urban or rural) and residence in a long-term care facility

Regarding the contribution of each variable, age played an important role in gender health care differences for hospital and ICU admission. Socioeconomic level explained also a high portion of the gender health care gap in hospital admission and ICU admission, especially in waves 4–7 (30.1% in hospital admission and 43.9% in ICU admission). It was also an important factor explaining gender gaps in the length of ICU stay in wave 1 (56.1%) and waves 2–3 (41.6%) and for the time from diagnosis to hospital admission across all the waves. Residing in a LTC facility explained a high proportion of gender health care differences for ICU admission, time to diagnosis, time to hospital admission and length of hospital stay, especially during the first wave. Finally, differences in morbidity burden were only important to explain gender differences in the length of hospital stay across all the period analysed (Supplemental Figs. 1, 2 and 3).

## Discussion

Men and women with COVID-19 infection had different profiles: women were generally older than men, had lower socioeconomic status, lived in LTC facilities more frequently and had a higher morbidity burden. Women also had a higher frequency of COVID-19 infection than men.

The health care received throughout the pandemic differed between men and women, with gender inequalities being observed. Women were diagnosed earlier than men, but these differences disappear in the last waves of the COVID-19 pandemic. Women were admitted to hospital and ICU less frequently than men and their stays were also shorter. Overall, these differences narrowed throughout the pandemic, but persisted for all the period analysed, even after adjusting for other characteristics such as age, socioeconomic status, morbidity burden or the patient's place of residence. Differences in sociodemographic characteristics and morbidity between women and men could explain partially the gender differences found, mainly in the later phases of the pandemic, but not in the first waves. Particularly striking is the importance of residing in an institution in the early phases and the high importance of socioeconomic status throughout the pandemic as explanatory factors for the gender inequalities found.

As widely described, the incidence of COVID-19 in women was higher than in men [15]. There are a number of circumstances that have been associated with these gender differences, such as genetic or hormonal factors [16], but also other factors that generate greater vulnerability in women, such as their lower socio-economic status [17].

Gender inequalities in health care have been observed. Men are admitted more often to hospital and ICU and stay longer in hospital, which results in a greater therapeutic effort. This phenomenon is well known and, unfortunately, is not limited to health crises. It has already been observed that, facing the same health problem, hospitalization rates are generally lower in women than in men [18, 19] which suggest that women could face more obstacles in accessing health care. So, women tend to stay at home, rather than being hospitalized and the length of hospital stay is generally shorter [20], which could be explained by their caring role, as it has been already described [21]. But there are other factors that may be involved in gender inequalities in health care, like health attitudes, health behaviours and health care needs [22]. In the context of the COVID-19 pandemic there are also other circumstances that may have contributed to gender inequalities. Men are usually cared by women in their homes but women, often older, are referred to nursing homes when they are ill. Women living in LTC facilities are in a situation of greater vulnerability, which increases if they also suffer from dementia, which has been associated in this context with a higher risk of not being admitted to hospital [23]. The greatest impact of the COVID-19 pandemic on institutionalized people has been associated with physical and psychological factors, living conditions and deficient policy responses [24–26], which seems to be associated with difficulty in accessing health care. Finally, women have lower socioeconomic status than men, a factor classically associated with poorer health care attention [27, 28].

Differences in all indicators of access to and use of health services are greater at the beginning of the pandemic and tend to decrease as the pandemic progresses, but do not disappear completely. These results show that, in times of health system crisis and great uncertainty, existing inequalities are exacerbated. In the case of COVID-19, the lack of clear protocols for action and a limited definition of the disease in the early stages of the pandemic that did not take gender differences into account may explain these greater inequalities in utilisation [29]. Moreover, as some authors point out [29], hospital and ICU admission are indicators of the severity of the disease, but also of the diagnostic and therapeutic effort required. In our study, and as it has been described in the literature [30], men had higher mortality rates than women. However, when we described the use of health services only in patients who died within 30 days of diagnosis, the gender inequalities in the use of services remained (results not shown). In these sub-analyses, men also showed a higher frequency of hospital admission than women, for all three time points analysed. Thus, in wave 1, men who died within 30 days of COVID-19 diagnosis had a hospital admission in 76.7% of cases, while in women this percentage was 69.6% (*p* = 0.031). The length of hospital stay was also longer in men, with statistically significant differences for the three time points analysed. Finally, admission to the ICU was also more frequent in men than in women, with the greatest differences in wave 1 (10.7% in men vs. 3.6% in women). This fact has been already observed in Spain, where the probability of admission to the ICU was higher in deceased men than in deceased women, which could indicate, among others, a greater therapeutic effort in men than in women, implying a gender bias in health care [31].

In addition, it should not be overlooked that some authors point to a higher percentage of undiagnosed excess COVID-19 mortality in women, related with an underreporting of COVID-19 deaths among women who died in LTC facilities, especially during the first wave [32], a higher frequency of deaths in women from other causes consistent with COVID-19 [31] and a greater hospital access and care in men [33] that have implied higher reporting of deaths in men in the pandemic. This aspect requires further attention and underlines the need for a gender-sensitive definition of the disease and for sex-disaggregated information in future pandemics [34].

In the first pandemic waves, especially in the first wave, when gender inequalities are greater, these inequalities are not justified either by sociodemographic factors or by morbidity factors of the subjects. As mentioned above, the confluence of factors such as older age, greater morbidity burden, lower socio-economic status and residence in a LTC facility explain, at least partly, the differences found between men and women in the later phases of the pandemic, but not at the beginning of the pandemic. Several factors could explain these differences between the early and late phases of the pandemic. Confinement may have made it particularly difficult for women to seek medical care, due to the burden it entails, which may have prevented them from seeking medical assistance [35]. There were also situations of gender-based violence, in which women did not seek medical care [8, 36]. Finally, the great heterogeneity of existing protocols in the initial phases may have increased the existing gender bias [4].

This study has some limitations. The sex disaggregation available in the electronic registers is binary in nature, which does not necessarily equate to the sex of the subject, and does not allow for the identification of persons with diverse gender identities. Unfortunately, this is the only information available. There are also some limitations inherent to observational studies, such as the existence of incomplete information. In this sense, quality of the data may have changed across the waves. The cause of hospital admission was not available. In order to solve this problem, a range from -15 to 30 days from hospital admission to COVID-19 diagnosis was applied. Some of the patients living in LTC facilities who were not hospitalized could have been treated in one of the “COVID centers” set up in Aragón in the first waves of the pandemic. Unfortunately, this information is not available. Other information not available was the severity of the infection. This aspect is relevant as it could have conditioned the use of health services by patients, mainly aspects such as the decision to be hospitalized or to be admitted to the ICU. However, as noted above, we replicated our analyses only in patients who died within 30 days of diagnosis, as a proxy for high-severity COVID-19 cases, with results similar to those in the general population. Finally, it is important to note that what is statistically significant is not always clinically relevant, and further studies are needed to understand the medical implications of the observed inequalities in health care utilization. On the other hand, this work has many strengths. This is a populational study based on a European population of 1.3 million people. We used data from administrative health data sources combined with electronic health records.

## Conclusions

Access to and use of health services during the COVID-19 pandemic has shown gender inequalities. These inequalities were greater in the first waves of the pandemic, but did not disappear. Differences between men and women could not be explained in the first waves by socio-demographic factors or by the morbidity burden of the patients, showing a different therapeutic effort. Moreover, the lack of clear protocols in a context of great uncertainty and health crisis seems detrimental to the equitable use of health services and may lead to less therapeutic effort in groups in vulnerable circumstances.

It is the task of public administrations to promote equity in accessibility and in therapeutic effort, in all aspects, including care, regardless of gender, in order to avoid inequalities in health. These differences are striking, as there is a legal regulation in Spain to avoid them [37]. On the other hand, it should be noted that inequalities in health are mainly due to the social determinants of health [38]. And although the main axes of inequality seem to be at the same level and to operate independently, it is necessary to study how the different opportunities of women and men interact with their socio-economic position, educational level or origin from an intersectional perspective.

Therefore, in order to reduce health inequalities between women and men, health research must take into account an intersectional gender perspective, which considers all risk factors and discriminatory factors that put women in a vulnerable situation [39]. Clear gender-sensitive guidelines, gender-sensitive definitions of disease, or provide appropriately disaggregated information, are essential to ensure equitable and quality health care. In addition, the inclusion of a gender perspective in decision-making processes is crucial for effective crisis response and recovery [40]. In this regard, efforts must be made to prepare for future pandemics and health crises by focusing on care for the most vulnerable groups, to ensure that existing inequalities are not exacerbated.

### Supplementary Information

**Supplementary Material 1.**

## Data Availability

No datasets were generated or analysed during the current study.

## Abbreviations

COPD: Chronic obstructive pulmonary disease
LTC: Long-term care
ICU: Intensive care unit
OR: Odds ratio
95%CI: 95% Confidence interval
