# Supplementary material for: Gender health care inequalities in health crisis: when uncertainty can lead to inequality
Source: Arch Public Health. 2024 Apr 2;82:46. doi: 10.1186/s13690-024-01276-7 (PMC10985974; doi:10.1186/s13690-024-01276-7)
Supplement: Supplementary file 1 — Supplementary Material 1. [file 13690_2024_1276_MOESM1_ESM.docx]

**SUPPLEMENTAL MATERIAL**

**Supplemental figure 1. Contribution of each variable to the explained decomposition of gender inequality in health care delivery in wave 1. Oaxaca decomposition analyses.** **

ICU: intensive care unit; LTC: long-term care. Bars shows the proportion of the gender gap explained by each variable.

**Supplemental figure 2. Contribution of each variable to the explained decomposition of gender inequality in health care delivery in waves 2 and 3. Oaxaca decomposition analyses.**

**

ICU: intensive care unit; LTC: long-term care. Bars shows the proportion of the gender gap explained by each variable.

**Supplemental figure 3. Contribution of each variable to the explained decomposition of gender inequality in health care delivery in waves 4 to 7. Oaxaca decomposition analyses.**

**

ICU: intensive care unit; LTC: long-term care. Bars shows the proportion of the gender gap explained by each variable.
